# The ‘lifecycle’ of human beings: a call to explore vector-borne diseases from an ecosystem perspective

**DOI:** 10.1186/s40249-020-00653-y

**Published:** 2020-04-16

**Authors:** Olav T. Muurlink, Andrew W. Taylor-Robinson

**Affiliations:** 1 Centre for Sustainable Innovation, School of Business & Law, Central Queensland University, Brisbane, QLD Australia; 2 Infectious Diseases Research Group, School of Health, Medical & Applied Sciences, Central Queensland University, Brisbane, QLD Australia

**Keywords:** Vector-borne disease, Dengue, Mosquito, Climate change, Ecosystem, Design thinking, Social innovation, Wicked problem

## Abstract

**Background:**

Dengue virus, an *Aedes* mosquito-borne flavivirus, is associated with close to 400 million reported infections per annum worldwide. Reduction of dengue virus transmission depends entirely on limiting *Aedes* breeding or preventing adult female contact with humans. Currently, the World Health Organization promotes the strategic approach of integrated vector management in order to optimise resources for mosquito control.

**Main text:**

Neglected tropical disease researchers focus on geographical zones where the incidence of clinical cases, and prevalence of vectors, are high. In combatting those infectious diseases such as dengue that affect mainly low-income populations in developing regions, a mosquito-centric approach is frequently adopted. This prioritises environmental factors that facilitate or impede the lifecycle progression of the vector. Climatic variables (such as rainfall and wind speed) that impact the vector’s lifecycle either causally or by happenstance also affect the human host’s ‘lifecycle’, but in very different ways. The socioeconomic impacts of the same variables that influence vector control impact host vulnerability but at different points in the human lifecycle to those of the vector. Here, we argue that the vulnerability of the vector and that of the host interact in complex and unpredictable ways that are characteristic of (complex and intransigent) ‘wicked problems’. Moreover, they are treated by public health programs in ways that may ignore this complexity. This opinion draws on recent evidence showing that the best climate predictors of the scale of dengue outbreaks in Bangladesh cannot be explained through a simple vector-to-host causal model.

**Conclusions:**

In mapping causal pathways for vector-borne diseases this article makes a case to elevate the lifecycle of the human host to a level closer in equivalence to that of the vector. Here, we suggest value may be gained from transferring Rittel and Webber’s concept of a wicked (social) problem to dengue, malaria and other mosquito-transmitted public health concerns. This would take a ‘problem definition’ rather than a ‘solution-finding’ approach, particularly when considering problems in which climate impacts simultaneously on human and vector vulnerability.

## Background

For well over a decade the World Health Organization (WHO) has advocated integrated vector management (IVM) as a means of combatting transmission of malaria, dengue and other mosquito-borne human pathogens of global significance. IVM is defined as “a rational decision-making process for the optimal use of resources for vector control” [1]. This focus is predicated on the assumption that what is good for the vector is bad for the host. This rationale governs a public health response that targets the lifecycle of the vector — for example, the timing of hatching of larvae, drainage of reservoirs of still water and residual spraying of insecticides inside domestic dwellings. This mosquito-centric approach has proven effective yet tends to de-emphasise the lifecycle of the host. The human ‘lifecycle’ is a vastly longer and more complex timeframe than that of the insect to which it plays host. During the human lifespan, vulnerability to vector-borne diseases (VBDs) fluctuates in response to more or less predictable changes in the local environment, such as harvest failure. A vector-centric approach may well mask causal relationships in the opposite direction: what is harmful to human health may benefit the mosquito vector in promoting transmission of disease.

Neglected tropical disease (NTD) researchers are predisposed to focus on geographical zones where the incidence of clinical cases is high. Logic suggests that these are regions in which the population density of vectors is concomitantly high. Importantly, it is no coincidence that NTD hotspots tend to be in developing countries characterised by socioeconomic constraints which are often overlooked when mapping VBD patterns. Using the human arboviral disease dengue as an exemplar, we argue that conditions which allow vectors and hosts to prosper are different in important ways that interact to complicate prediction of the spread of VBDs.

## Main text

### The growing public health threat of dengue

With 390 million reported infections per annum, 96 million symptomatic cases per annum [2], at least 500 000 hospitalisations [3], and approximately 22 000 fatalities [4], dengue ranks as a highly significant global VBD. While clinical infection typically manifests as an uncomplicated, non-specific febrile illness (dengue fever), in a minority of patients this progresses to the life-threatening dengue haemorrhagic fever or dengue shock syndrome. At present, there is no specific anti-dengue therapy although a currently controversial vaccine has very recently received approval from the United States Food and Drug Administration for prophylactic use [5]. The current non-availability of an efficacious antiviral drug or universally licensed vaccine and a lack of effective vector control strategies combine to make dengue a serious public health scourge [6].

The aetiological agent of infection is dengue virus (DENV), of which there are four established serotypes. While humans are the principal host some DENV serotypes also infect non-human primates such as macaques. Evidence points to *Aedes aegypti*, the yellow fever mosquito, and *Ae. albopictus*, the Asian tiger mosquito, both day-biting, as the major vectors for dengue transmission around the world [7].

Dengue is endemic to more than 125 countries in tropical and subtropical zones of the world [6]. Asia, South America and the Pacific Islands are hyper-epidemic regions — like Bangladesh, all with significant and stubborn pockets, not just of the vector’s existence but also that of human poverty and population vulnerability [8].

### Disease outbreak predictors have lag times inexplicable by vector-centric thinking

Ourselves and colleagues recently conducted a near-exhaustive data mining analysis of relationships between climatic variables and dengue in Bangladesh [9]. The best predictors of an outbreak of dengue are not surprisingly assumed to be environmental conditions that benefit *Aedes* vector species, which have a short lifecycle of up to 3 weeks. Conditions that cause the vector to thrive should in turn be associated with the threat of VBDs. We focused on the two hotspots of dengue infection within Bangladesh, Chittagong and Dhaka [9]. By holding constant the number of rainy days in the month prior to an outbreak for each degree Celsius increase in temperature, the risk of an outbreak (defined as at least one confirmed case of dengue at a local clinic) increased by 23%. However, the best predictor of the scale of an outbreak (defined as the number of patients diagnosed with the disease), was much more distal in time — the percentage average humidity six months prior to the outbreak. The relationship was statistically highly significant (*P* < 0.0001) but, in absolute terms, weak (explaining ‘just’ 15% of variance). It was, however, the strongest predictor of the scale of an outbreak. Six months is beyond the timescale that can be explained by the lifecycle of *Aedes* mosquitoes but can be much more readily interpreted by reference to variables relevant to the vulnerability of the human host.

### Nutrition and weather as distal predictors of disease outbreak

Rice is a key dietary staple in many developing countries, including Bangladesh. Here, sub-economies of the financially impoverished prosper whenever the rice harvest flourishes and market prices drop. While the economy is in constant transition the proportion of employed labour that is allocated to the agriculture, forestry and fishery sector remains stubbornly close to 50% [10]. Thus, essentially half the population is reliant on an industry that is directly dependent on vagaries of the weather, and for these families even seasonal variation in birthweight can be observed, indicating sensitivity to external conditions [11]. The centrality of rice to the diet and to the nation’s economy thus brings the lag time between crop failure and undernutrition in vulnerable communities to the fore.

Despite a plethora of options available from the Bangladesh Rice Research Institute (BRRI), in practice the cultivation of Asian rice (*Oryza sativa*) in Bangladesh is concentrated on just two varieties, BRRI Dhan 28 and BRRI Dhan 29. BRRI Dhan 28 is regarded as a ‘short maturity duration’ crop with a days-to-flowering range of 81–115 depending on seasonal variations [12]. For BRRI Dhan 29 the gestation period between planting and harvest is typically around 30 days longer [13]. In this context, therefore, the 6 months predictor between climatic events and the scale of dengue outbreaks makes eminent good sense. Climatic changes that directly compromise rice harvests, and therefore the nutrition status of people for whom this cereal grain is a staple food, also indirectly impair functioning of the immune system [14]. Hence, a reduced rice crop may compromise immune responses to dengue infection in vulnerable populations — several months later. Thus, unfavourable weather conditions in which to grow rice, a subsequent poor harvest and resultant undernutrition among locals may each be considered as a surrogate marker of impaired immunity to dengue in an endemic area. That susceptibility needs to be included in a systems analysis of VBD was raised in an early discussion of climate change and emerging infectious diseases [15] and our study reinforces that call.

Further research is required to determine if the mediating factor between relative humidity 6 months before a dengue outbreak and its scale of incidence is valid or spurious, but clearly a simple vector-focused explanation is inadequate. Muurlink et al. [9] demonstrate that this effect is *not* merely caused by climate predicting climate. We acknowledge that data mining analyses inherently generate a risk of the emergence of ‘false negative’ relationships, masked behind statistical significance. Notwithstanding, the study does provide a useful reminder that climate change may follow multiple paths to impact on human health and wellbeing.

### Rethinking vector-borne diseases via a social innovation approach

The concept of the ‘magic bullet’ in medicine may have originated with Ehrlich’s pursuit of a cure for syphilis, caused by the sexually-transmitted bacterium *Treponema pallidum* [16]. However, the view that a disease can be tackled with minimal regard for context persists over a century later and still remains a translational research target [17]. Developed as a creative technique for visual designers, ‘design thinking’ was only relatively recently repurposed to the task of tackling ‘wicked problems’, issues that are highly (if not intractably) complex [18]. Rittel and Webber’s classic treatise [19] defines wicked problems in a number of ways, including those that are complex in aetiology, frequently nested inside another problem, subject to recursive causal loops and historically resistant to a solution. From the perspective of clinical diagnosis, prevention and treatment, most major medical challenges can be regarded as wicked, so could be subject to analysis through a form of design thinking. Expressed simply, design thinking about wicked problems attempts to deploy both systems *and* individual perspectives, in order to explore the nature, or the ‘design’, of a problem.

Indeed, Buchanan’s influential early work in the field [18] suggests we should adopt a ‘problem definition’ approach to overcoming the problem. Understanding intimately how a problem has arisen, over a timespan of possibly decades, and how the problem has evolved to compensate for measures designed to overcome it, may seem an act of extreme anthropomorphism. However, such an approach helps to illuminate why a problem is complex and resistant to ‘simple’ interventions that fail to consider its fluid ‘ecosystem’ [20]. For the purposes of this discussion an ecosystem is defined as a geographically confined cluster of interacting living and non-living elements.

**Fig. 1:**
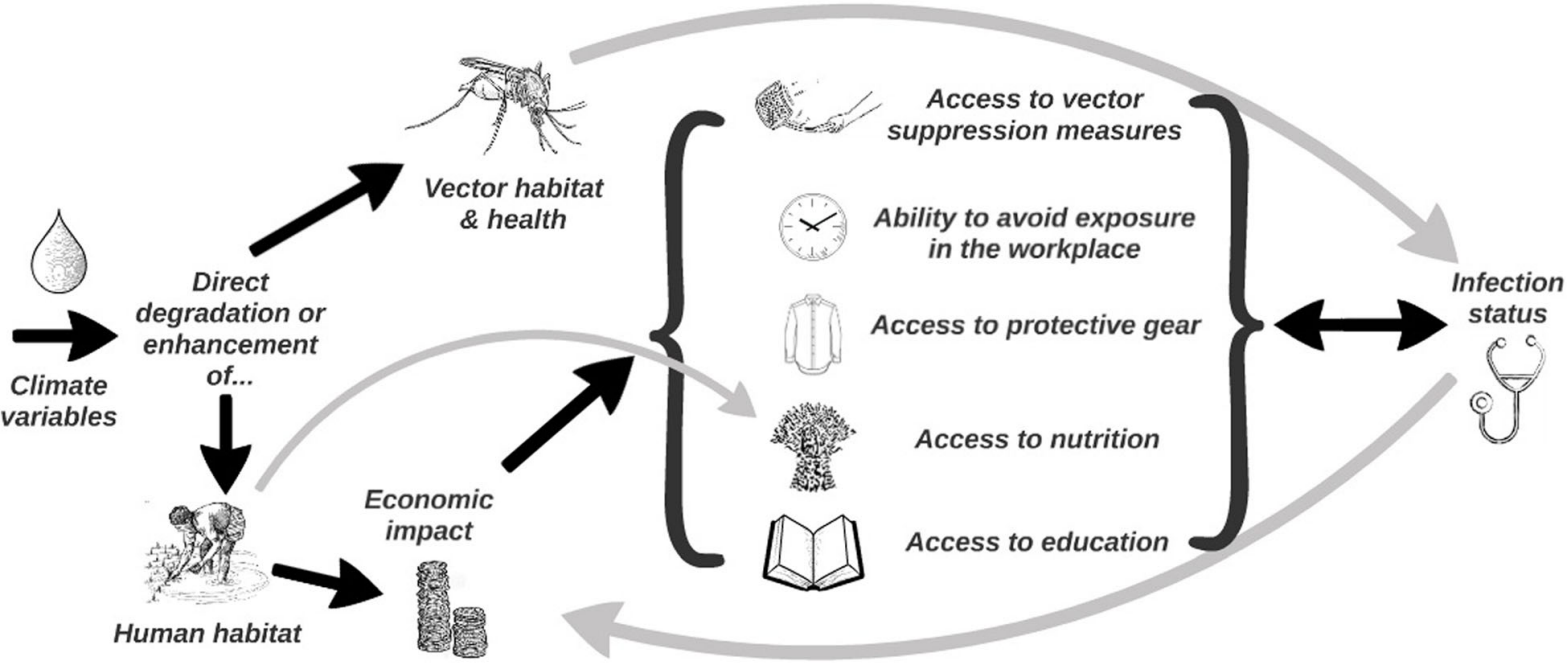
A simple schematic of different pathways between climate and disease, highlighting how both social and vector elements interact

Such an ecologically framed perspective to understanding VBDs should foreground the complex ‘lifecycle’ of the human being, *Homo sapiens* as well as that of the vector. A lifecycle approach captures different stages of life: pregnancy, birth, infancy, the toddler years, childhood, puberty, older adolescence, adulthood, middle age, and the senior years. Throughout these stages, principally by virtue of differences in immune system development and integrity [21] and degree of exposure [22], individual variation in susceptibility to VBDs is apparent [23], although this may be masked at a population level [24]. While vulnerability remains a contested and ambiguous construct [25], it is also considered as a useful analytical tool to help explain otherwise aberrant or paradoxical findings [26]. In their commentary on global health multipliers, Stuckler, McKee and Basu urge researchers to “look upstream” at social determinants of disease [27]. This is not uncommon in relation to diseases that have a *prima facie* human social and cultural context such as HIV/AIDS [28]. Yet, for a VBD that arises because of a (clearly recognisable) ‘attack’ by a vector, the temptation to examine the systems nature of the disease may be reduced. So, we tend to interpret climate impacts on VBD through their direct effect on the ability of the vector to thrive. However, the vulnerability of both vectors and humans to meteorological variables interacts to produce a problem that is more complex and arguably even wicked (Fig. 1).

Precipitation and air movement impact a mosquito’s ability to prosper in its habitat. For humans, however, such climate variables more commonly flow through socioeconomic pathways. For example, these can determine the type and standard of buildings people are able to construct, the local availability of insect repellents and a community’s access to affordable, high quality health care or even to education on control and prevention.

Such multifactorial interactions between wealth and health, or more importantly between socioeconomic factors and vulnerability, will not always play out in a predictable fashion. As Fig. 1 indicates, lower wealth may be associated with greater vulnerability — for example reduced access to nutrition, education and protective measures, but also reduced ability to adjust (for instance) work hours in order to avoid exposure — but greater wealth can also lead to greater vulnerability. An illustration of this latter, less commonly acknowledged association in VBD studies is a recent investigation of malaria trends in Uganda that revealed a causal relationship between electrification of households (an indicator of socioeconomic status) and incidence of clinical infection [29]. These authors suggested that by attracting *Anopheles* mosquitoes the use of domestic electric lights and outdoor night lighting may inadvertently increase the exposure of humans to vectors of malaria transmission. Notably, this might have the unwanted effect of artificially extending the period during which the vector is most active and thus receptive to taking a human blood meal. A number of researchers, for example [30], have identified the possibility that causation pathways between low socioeconomic status and VBDs travel in both directions.

## Conclusions

Of itself, the finding that climatic fluctuations can impact on the reported incidence of dengue is clinically important. The intensity of research focus on climate change has driven examination of a broad suite of health impacts. Global warming is bringing about alterations in meteorological patterns (including volume and timing of rainfall). Seemingly small changes in complex systems, such as those involved in weather, may thus have statistically significant implications for human health, while making an equally profound impact on the health of vector mosquito species [31].

The WHO’s IVM approach to treating VBDs pivots on controlling the vector. Its 78-page handbook [1] touches on management of the vector but uses the term “vector control” far more frequently — 215 times — than it refers to the need to understand the lifecycle of the vector or of the host. This quintessential reference guide supports an ecosystem approach to understanding “identifying the diversity and habitats of vector species” (p. 25) but only in the cause of vector control. Perhaps most usefully, it refers to “vector control needs assessment” as covering socio-political issues such as policy, human resource management and education, yet still positions the vector in an oppositional relationship with humans.

In the language of the emerging discipline of social innovation, the WHO’s IVM strategy may be conceptualising what is in fact a wicked problem as a relatively simple one. More than 20 years ago Patz et al. [15] urged interdisciplinary collaboration among climatologists, social scientists and medical researchers in order to aim to understand the role of climate variables in the emergence of human infectious diseases. It is arguably time to reconsider how to combat dengue and other major vector-borne NTDs from an ecosystem perspective. Research on *vulnerability* to dengue is expanding rapidly. Currently, this focuses primarily on spatial distributions of vector species relative to human hosts, for example [32], but a new research agenda drawing on socio-economic and nutritional variables is emerging.

## Data Availability

Not applicable.

## Abbreviations

WHO: World Health Organization
IVM: Integrated vector management
VBD: Vector-borne disease
NTD: Neglected tropical disease
DENV: Dengue virus
BRRI: Bangladesh Rice Research Institute
